# RETRACTION: Effect of Surface Modification of Nanofibres with Glutamic Acid Peptide on Calcium Phosphate Nucleation and Osteogenic Differentiation of Marrow Stromal Cells

**DOI:** 10.1155/term/9820625

**Published:** 2026-06-26

**Authors:** Journal of Tissue Engineering and Regenerative Medicine

RETRACTION: O. Karaman, A. Kumar, S. Moeinzadeh, X. He, T. Cui, and E. Jabbari, “Effect of Surface Modification of Nanofibres with Glutamic Acid Peptide on Calcium Phosphate Nucleation and Osteogenic Differentiation of Marrow Stromal Cells,” *Journal of Tissue Engineering and Regenerative Medicine* 10, no. 2 (2016): E132–E146, https://doi.org/10.1002/term.1775.

The above article, published online on 30 July 2013 in Wiley Online Library (https://wileyonlinelibrary.com), has been retracted by John Wiley & Sons, Ltd, following concerns raised by a third party. An investigation identified several instances of duplication of elements within Figures 5 and 8. The authors cooperated with the investigation; however, due to the time elapsed since publication, they were unable to provide the original raw data. While some supporting data were provided, this was not sufficient to restore confidence in the article’s results and conclusions. Therefore, the publisher considers this article unreliable. The authors disagree with the retraction.

